# Exploration of Potential Diagnostic Value of Protein Content in Serum Small Extracellular Vesicles for Early-Stage Epithelial Ovarian Carcinoma

**DOI:** 10.3389/fonc.2021.707658

**Published:** 2021-09-15

**Authors:** Pu Li, Yuezong Bai, Boer Shan, Wei Zhang, Zhanjie Liu, Yingjie Zhu, Xiaoya Xu, Qian Chen, Xiujie Sheng, Xiaoyang Deng, Zhengchen Guo, Dadong Zhang, Huaying Wang, Yanan Zhang, Yuanjing Hu

**Affiliations:** ^1^Department of Gynecology Oncology, Tianjin Central Hospital of Obstetrics and Gynecology, Tianjin, China; ^2^3D Medicines Inc., Shanghai, China; ^3^Department of Gastrointestinal Oncology, Peking University Cancer Hospital & Institute, Beijing, China; ^4^Department of Gynecologic Oncology, Fudan University Shanghai Cancer Center, Shanghai, China; ^5^Department of Gynecology, Yunnan Tumor Hospital, The Third Affiliated Hospital of Kunming Medical University, Kunming, China; ^6^Key Laboratory for Major Obstetric Diseases of Guangdong Province, Gynecology Department of the Third Affiliated Hospital of Guangzhou Medical University, Guangzhou, China; ^7^Gynecology Department, The First Affiliated Hospital of Chengdu Medical College, Chengdu, China

**Keywords:** early diagnosis, epithelial ovarian carcinoma, multi-center population-based study, protein contents, serum, small extracellular vesicle

## Abstract

Epithelial ovarian carcinoma (EOC) is one of the most common gynecologic malignancies with a high mortality rate. Serum biomarkers and imaging approaches are insufficient in identifying EOC patients at an early stage. This study is to set up a combination of proteins from serum small extracellular vesicles (sEVs) for the diagnosis of early-stage EOC and to determine its performance. A biomarker for early-stage ovarian cancer (BESOC) cohort was used as a Chinese multi-center population-based biomarker study and registered as a Chinese Clinical Trial ChiCTR2000040136. The sEV protein levels of CA125, HE4, and C5a were measured in 299 subjects. Logistic regression was exploited to calculate the odds ratio and to create the sEV protein model for the predicted probability and subsequently receiver-operating characteristic (ROC) analysis. The combined sEV marker panel of CA125, HE4, and C5a as a sEV model obtained an area under curve (AUC) of 0.912, which was greater than the serum model (0.809), by ROC analysis to identify EOC patients from the whole cohort. With the cutoff of 0.370, the sensitivity and specificity of the sEV model were 0.80 and 0.89, which were much better performance than the serum markers (sensitivity: 0.55~0.66; specificity: 0.59~0.68) and the risk of ovarian malignancy algorithm (ROMA) index approved by the U.S. Food and Drug Administration (sensitivity: 0.65; specificity: 0.61), to identify EOC patients from patients with benign ovarian diseases or other controls. The sEV levels of CA125 significantly differed among early-stage and late-stage EOC (*p* < 0.001). Moreover, the AUC of ROC to identify early-stage EOC patients was 0.888. Further investigation revealed that the sEV levels of these 3 proteins significantly decreased after cytoreductive surgery (CA125, *p* = 0.008; HE4, *p* = 0.025; C5a, *p* = 0.044). In summary, our study showed that CA125, HE4, and C5a levels in serum sEVs can identify EOC patients at the early stage, elucidating the possibility of using a sEV model for the diagnosis of early-stage EOC.

## Introduction

Ovarian cancer is the leading gynecologic malignancy and the most common cause of gynecologic cancer death (1). Approximately 95% of primary ovarian malignancies originate from epithelial cells (2, 3). The prognosis of early-stage patients is satisfactory (the five-year survival rates of International Federation of Gynecology and Obstetrics (FIGO) stage I and II patients are 81.3% and 66.9%, respectively, and the five-year survival rates of FIGO stage III and IV patients are only 41.3% and 31.3%, respectively) (4). However, more than 60% of patients with ovarian cancer are diagnosed at advanced stages. The high mortality rate of epithelial ovarian carcinoma (EOC) can be attributed to the fact that the majority of patients are diagnosed with advanced disease (5). Therefore, an approach to identify EOC at an early, localized, and curable stage can significantly reduce mortality rate.

Unfortunately, attempts to detect EOC at an earlier stage using serum CA-125 and/or transvaginal ultrasonography (TVUS) have not been successful (6–8). Multiple studies have utilized serum CA-125 levels as a screening marker for ovarian cancer. Fifty to ninety percent of early EOC patients showed elevated CA-125 levels (9), but numerous other conditions can increase CA-125 levels (10, 11). The serum level of human epididymis protein 4 (HE4) showed higher sensitivity than CA-125 when identifying patients with ovarian cancer patients from patients with benign gynecologic disease (12). However, HE4 appears to differ due to multiple non-ovarian conditions such as pregnancy, menopause status, and rake (13–15). The secreted proteins of malignant cancer cells might be detected earlier; however, the signals of which are often masked by various proteins (e.g., albumin, immunoglobulin) in blood (16).

Small extracellular vesicles (sEVs) are bilayer membrane vesicles that contain proteins and nucleic acids, thus reflecting the contents of the cell from which these originate (17). Several studies have demonstrated the prominent roles of sEV in the progression of various cancers (18). Furthermore, sEVs can be isolated from plasma and serum to reduce the interference of other abundant plasma proteins (16). Therefore, the protein in or on top of sEVs can be a potential source of biomarkers for the detection of early-stage diseases. For example, Kalluri et al. reported that sEV covered with the proteoglycan glypican-1 may be a potential diagnostic tool for early-stage pancreatic cancer, with a sensitivity of 100% and specificity of 100% (19).

In a previous sEV proteomics study, the complement system is reported as one of the most over-expressed pathways in the sEVs of EOC patients (20). Complement component 5a (C5a), a core protein of the complement system, has been associated with the pathological status of EOC (21, 22). Having been detected in a sEV-proteomics study, we hypothesize that sEV levels of C5a may be potential utilized as a marker for EOC patients (20). In this study, we examined protein levels of sEV-derived CA125, HE4, and C5a as candidates of potential biomarkers for EOC, particularly at the early stages, in a larger and more complex Chinese cohort.

## Materials and Methods

### Study Design and Participants

The patients with ovarian cancer and benign ovarian disease in this study were enrolled in the Biomarker for Early Stage Ovarian Cancer (BESOC) cohort between 2016 and 2018. BESOC is a multi-center (n = 5, Tianjin Central Hospital of Gynecology Obstetrics, Fudan University Shanghai Cancer Center, the Third Affiliated Hospital of Kunming Medical University, the Third Affiliated Hospital of Guangzhou Medical University, and the First Affiliated Hospital of Chengdu Medical College) cohort (2016–2021) registered as a Chinese Clinical Trial ChiCTR2000040136 (http://www.chictr.org.cn/showproj.aspx?proj=63907) and enrolled subjects (over 20 years old) who presented an ovarian adnexal mass and went through surgery afterwards. To diversify the control group, gastric cancer or colorectal cancer patients were enrolled. The female patients with gastric cancer or colorectal cancer in this study were enrolled in the Gastrointestinal Cancer Cohort in 2017, which is a prospective, single-center (Changhai Hospital of Second Military Medical University) cohort that enrolled subjects (over 20 years old) who were diagnosed with gastrointestinal cancer. Blood samples and clinical information were collected on admission before cancer-related therapies (surgery, radiotherapy, or chemotherapy). All blood samples were collected in serum tubes and spun at 4,000 g for 10 min at 4°C to isolate the serum.

The healthy subject group consisted of age-matched healthy female volunteers (no diagnosis of any cancer, no family history of breast cancer or ovarian cancer, and ovarian-related-disease-free at least six months after sample collection), undergoing routine gynecologic examinations. The post-operation samples of 20 stage IIb or IIIc patients were collected 6 days after surgery (e.g., salpingo-oophorectomy, omentectomy, and pelvic and paraaortic lymph node dissection). All serum samples were stored at -80°C until analysis.

The selection of each subject was reviewed by two dedicated gynecologic pathologists, who were blinded to each other’s diagnosis and serum marker levels. The diagnosis and staging were decided based on the post-operation histopathology reports. Tumors were classified and divided into pathological subtypes: serous, mucinous, endometrioid, clear cell, and others. According to the FIGO classification criteria, all EOC patients were diagnosed as stage I to stage IV (23). In this study, early stages included stages I and II, and advanced stage included stages III and IV. A flowchart of this study is shown in **Figure S1**.

### Isolation of Serum sEVs

The sEV isolation method was previously reported in detail (24) and strictly followed in this study. Briefly, 3D-EVN kit (3DMed Co., Ltd., #3DEVN3525, China Food and Drug Administration Ref. No.# SHMHMD20170019, v/v, 1:4) was added to 1 mL of serum and mixed until cloudy. The mixture was spun at 4,700 g at 4°C for 10 min. The pellet was lysed in 100 µL of a 3D-EVL lysis kit (3DMed Co., Ltd., #3DEVL0409) and used as 10-fold concentration to meet the detection range of the subsequent immunoassay.

### Characterization of Serum sEVs

To characterize of the serum sEVs, western blotting (WB), nanoparticle tracking analysis (NTA), and electron microscopy (EM) were performed in this study. To carry out the EM analysis, the isolated sEVs were resuspended in PBS and fixed in 5% glutaraldehyde. After washing with PBS for 5 min, the sEVs were immobilized in 1% OsO_4_ in PBS and dehydrated with a series of ethanol concentrations (40%, 60%, 80%, and 96–98%). After the ethanol was evaporated, the samples were allowed to dry at ambient temperature for 24 h on Si substrate and then analyzed *via* EM (Hitachi High-Technologies, Tokyo, Japan) after gold-palladium sputtering. To perform the WB, the extraction of sEV proteins was done using the 3D-EVN kit (3DEVN3525; 3DMed, Shanghai, China) and sEVs were homogenized in RIPA lysis buffer with proteinase inhibitors (P0013B; Beyotime, Shanghai, China) on ice for 30 min. Then the lysed samples were centrifuged at 12,000 × g for 10 min at 4°C, and the protein concentration of the supernatant was measured using the Pierce™ BCA Protein Assay Kit (Thermo Fisher Scientific, USA). The anti-Alix antibody (diluted 1:1000; cat. no. 2171; Cell Signaling Technology, Danvers, MA, USA), anti-CD9 antibody (diluted 1:500; cat. no.13,174; Cell Signaling Technology, Danvers, MA, USA) and anti-TSG101 polyclonal antibody (diluted 1:500; cat. no. abs115706; Absin Bioscience Inc., Shanghai, China) were used as the primary antibodies. Horseradish peroxidase-conjugated goat anti-rabbit IgG and goat anti-mouse IgG antibodies (Beyotime Biotechnology, China) were used as the secondary antibodies. The antibody binding was detected using an enhanced chemiluminescence system according to the manufacturer’s protocol (Tanon-5200 Multi; Tanon Science & Technology Co. Ltd., Shanghai, China). For the particle size and concentration of serum sEVs, a Nanosight NS 300 system (NanoSight Technology, Malvern, UK) was used. Each sample was configured with a 488-nm laser and a high-sensitivity scientific complementary metal-oxide semiconductor camera, and measurement were performed in triplicate at camera setting 13 with an acquisition time of 30 s and a detection threshold setting of 7. At least 200 completed tracks were analyzed and obtained per video. Finally, the NTA analytical software (version 2.3) was used to analyze the nanoparticle tracking data of serum sEV samples.

### Human Protein Level Measurement and ROMA Calculation

The levels of CA125 II and HE4 were measured by Cobas e 602 analyzer (Roche Diagnostics) with corresponding assays (Roche Diagnostics # 11776223 for CA125 II in U/mL and # 05950929 for HE4 in pmol/L) based on standard protocols (ISO15189:2012). The level of EV C5a levels were measured with ELISA (R&D Systems #DY2037, in ng/mL). All tests were run in duplicates.

In this study, the cut-offs of serum levels of CA125, HE4, and the risk of ovarian malignancy algorithm (ROMA) were adapted for the Chinese population based on the results of the clinical trial of Tian et al. (15). The cutoff of serum CA125 II levels was 35 U/mL (15). The cutoff for serum HE4 levels was 105.10 pmol/L for the overall Chinese population, 68.96 pmol/L for the premenopausal population, and 114.90 pmol/L for the postmenopausal population (15).

Based on the clearance of Roche Diagnostics from the Food and Drug Administration (FDA 510(k) #K153607), ROMA was calculated using the following algorithms:

*Premenopausal: PI* = -12.0 + 2.38 × LN(*HE4*) + 0.0626 × *LN*(*CA*125); and
*Postmenopausal: PI* = -8.09 + 1.04 × LN(*HE4*) + 0.732 × *LN*(*CA*125).

Menopause was defined as 12 months without a menstrual period.

*ROMA Calculation Tool using Elecsys® assays value = Exp*(*PI*)/(1 + *exp*(*PI*)) × 10.

The index of ROMA ≥ 1.14 and ≥ 2.99 indicates a high likelihood for the presence of epithelial ovarian cancer in premenopausal and postmenopausal women, respectively (25).

### Statistical Analyses

Differences among groups (*p*-values) were analyzed using the Chi-square test for categorical variables, T-tests for normally distributed continuous variables, and Mann-Whitney-U-tests for continuous variables that were not normally distributed. The pre-/post operation comparison was conducted with using a paired t-test. To calculate the odds ratio (OR) and enable the direct comparison among variables, protein levels were converted into standard deviation units or z-scores using the observed value minus the mean value and divided by the standard deviation. Natural logarithm transformed values were used to reduce the effect of skewness in the distribution of protein levels. Differences with a *p*-value less than 0.05 were deemed statistically significant.

To assess the sensitivity and specificity of the serum sEV model, pathological diagnosis is regards as gold standard. In the EOC diagnosis: sensitivity = true positive/(true positive + false negative); specificity = true negative/(true negative + false positive); positive predictive value (PPV) = true positive/(true positive + false positive)*100%; negative predictive value (NPV) = true negative/(true negative + false negative) *100%.

Logistic regression was exploited to calculate the odds ratio and to create the sEV protein model for the predicted probability and subsequently receiver-operating characteristic (ROC) analysis. Both combined serum maker model and combined sEV protein model were built by entering the corresponding variables. All of the statistics were conducted with SPSS^®^ (IBM^®^, version 24.0.0.0).

## Results

### Baseline Characteristics

In this study, 299 subjects were enrolled (**Figure S1**). In the event group, 117 patients were diagnosed as EOC without other gynecologic complications, and serous carcinoma was the most often diagnosed type (**Table 1**). A total of 50 (42.7%) patients were at stage I or II and 67 (57.3%) were at stage III or IV. Seventy-four patients with benign ovarian diseases, 54 apparently healthy subjects, and 54 patients with gastrointestinal cancer were used as controls. Patients in the EOC group were significantly more often taking aspirin (47% vs. 26%) within 3 months prior to sample collection.

**Table 1:** Baseline characteristics of study-cohort.

Variables (N = 299)		Control (N = 182)	EOC (N = 117)	*P*-value
Age* (yrs)		53.0 ± 12.1	52.0 ± 9.4	0.428
**Menopause (N = 250)**		123 (75.0%)	69 (80.2%)	0.431
**Medical history**				
	Intrauterine device	32 (24.4%)	8 (19.5%)	0.672
	Infertility	1 (0.5%)	0	1.000
	Menopausal hormone therapy	11 (10.0%)	7 (9.3%)	1.000
	Endometriosis	0	1 (2.4%)	0.238
**Medication within 3 months**				
	NSAIDs	17 (9.3%)	11 (9.4%)	1.000
	Combined oral contraceptive pills	11 (6.0%)	7 (6.0%)	1.000
	Progestins	7 (3.8%)	7 (6.0%)	0.412
	Aspirin	47 (25.8%)	55 (47.0%)	**<0.001**
	GnRH agonists/antagonists	6 (3.3%)	3 (2.6%)	1.000
**EOC FIGO Class**				
	I	NA	30 (25.6%)	
	II	NA	20 (17.1%)	
	III	NA	55 (47.0%)	
	IV	NA	12 (10.3%)	
**Pathological type**				
	serous	NA	47 (40.2%)	
	mucinous	NA	23 (19.7%)	
	clear cell	NA	17 (14.5%)	
	endometroid	NA	20 (17.1%)	
	Others	NA	10 (8.5%)	
**Benign ovarian diseases**		74 (40.7%)	NA	
	Ovarian cyst	21 (11.5%)	NA	
	Ovarian Cystadenoma	19 (10.4%)	NA	
	Endometriotic cyst	11 (6.0%)	NA	
	Mature teratoma	11 (6.0%)	NA	
	Others	12 (6.6%)	NA	
**Gastrointestinal cancer**		54 (29.7%)	NA	
	Colorectal cancer	19 (10.4%)	NA	
	Gastric cancer	19 (10.4%)	NA	
	Others	16 (8.8%)	NA	

All numbers are presented as N (%) for categorical variables. *Mean ± sd is shown for continuous variables. Statistically significant differences are shown in bold. EOC, epithelial ovarian carcinoma; NSAIDs, nonsteroidal anti-inflammatory drugs; GnRH, Gonadotropin-releasing hormone; FIGO, the International Federation lof Gynecology and Obstetrics; NA, not applicable.

### Characterization of Serum sEVs

EM, WB, and NTA results to qualify the minimum information for studies of extracellular vesicles 2018 (MISEV2018) (26) are shown (**Figure S2**). EM analysis of representative sample showed that serum small EVs isolated in this study were bowl-shaped (**Figure S2A**). In addition, the sEV protein markers Alix, TSG101 and CD9 were present in the six representative samples using WB (**Figure S2B**). The size distribution of serum sEVs showed a main peak around ~60 nm by NTA analysis (**Figure S2C**).

### Serum sEV and Serum Protein Levels Between the EOC and Control Groups

All of the measured protein levels in both serum sEVs and serum were significantly different between the EOC and control groups. The levels of C5a (OR 7.537, *p* < 0.001), CA125 (OR 27.413, *p* < 0.001), and HE4 (OR 69.973, *p* < 0.001) in the EVs were significantly higher in EOC patients compared to controls (**Table 2**). Although the serum level of CA125 and HE4 as well as ROMA index were also significantly higher in the EOC group (**Table 2**), at the corresponding cutoff points, the sensitivity of these 2 markers was 0.66 and 0.56, and specificity was 0.68 and 0.68, respectively (**Table 3**). Moreover, the sensitivity and specificity of the ROMA index were 0.65 and 0.61, respectively (**Table 3**). Notably, the odds ratios of serum sEV proteins were obviously higher than the serum markers.

**Table 2:** Protein levels from serum sEV and serum between the control and EOC groups.

Variables	Control	EOC	Odds Ratio	*P*-value
**Serum**				
CA125	23.65 (16.31, 47.70)^*^	102.7 (24.22, 372.00)	1.336	**<0.001**
HE4	87.20 (50.78, 113.70)	124.5 (73.60, 466.40)	5.875	**<0.001**
ROMA	2.17 (1.61, 3.11)	6.44 (1.88, 9.19)	3.028	**<0.001**
**sEV**				
C5a	8.31 (6.63, 11.66)	28.22 (11.70, 45.80)	7.537	**<0.001**
CA125	1.88 (1.13, 4.63)	41.11 (9.07, 144.58)	27.413	**<0.001**
HE4	2.95 (1.90, 3.83)	9.37 (4.50, 33.07)	69.973	**<0.001**

^*^All of the numbers are presented as the median (interquartile range). Statistically significant differences are shown in bold. EOC, epithelial ovarian carcinoma; sEV, small extracellular vesicles; ROMA, risk of ovarian malignancy algorithm.

**Table 3:** The sensitivity and specificity of serum markers and sEV model to identify the EOC patients from the whole cohort.

	Sensitivity	Specificity	PPV (%)	NPV (%)
serum HE4 (Overall)^*^	0.56	0.68	53	71
serum HE4 (pre-/post-menopause)^‡^	0.55	0.59	41	71
serum CA125^#^	0.66	0.68	57	75
ROMA^**^	0.65	0.61	47	83
sEV model (cut-off at 0.370)^^^	0.80	0.89	83	87

^*^the cut-off was at 105.10 pmol/L; ^‡^the cut-off of pre-menopause was at 68.96 pmol/L, post-menopause was at 114.90 pmol/L; ^#^the cut-off was at 35 U/mL; ^**^the cut-off of pre-menopause was at 1.14, post-menopause was at 2.99; ^^^the cut-off was at 0.370. PPV, positive predictive value; NPV, negative predictive value; ROMA, risk of ovarian malignancy algorithm; sEV, small extracellular vesicles.

### Serum sEV Proteins to Identify Early-Stage EOC Patients From Other Groups

After establishing that serum sEV protein levels are related to EOC, we then examined the potential of identifying early-stage patients. All of the protein levels were significantly higher in the late-stage group compared with the early-stage group (*p* < 0.05). However, serum CA125 levels or ROMA indices showed no difference among the early-stage, benign ovarian disease, and other cancer groups. Serum HE4 levels were significantly higher in the early-stage group than in the healthy and benign ovarian disease groups (**Table 4**). Serum sEV C5a levels were significantly lower in the healthy group compared to the early-stage group (*p* = 0.007), and almost showed a significant difference between the early-stage group and the benign ovarian disease and other cancer groups (*p* = 0.085 and 0.061, respectively). Meanwhile, serum sEV CA125 and HE4 levels significantly differed between the early-stage and any other group (**Table 4**). This implies the potential of serum sEV proteins to discriminate early-stage EOC patients from healthy subjects, benign ovarian disease patients, and other gastrointestinal cancer patients.

**Table 4:** Comparison of protein levels from serum sEVs and serum in early-stage EOC patients versus healthy subjects, late-stage EOC, benign ovarian disease, and gastrointestinal cancer patients.

	Early stage - Healthy^†^		Early stage - Late stage^†^		Early stage - Benign diseases^†^		Early stage - GI cancers^†^	
	mean difference^*^	*P*-value	mean difference	*P*-value	mean difference	*P*-value	mean difference	*P*-value
**Serum**								
CA125	141.27 ± 138.30*	0.221	-285.58 ± 132.83	**0.001**	24.13 ± 130.12	0.596	-107.69 ± 140.78	0.057
HE4	161.62 ± 51.48	**<0.001**	-230.21 ± 49.45	**<0.001**	98.47 ± 48.44	**0.017**	75.40 ± 52.41	0.222
ROMA	1.95 ± 0.49	0.139	-3.31 ± 0.49	**<0.001**	0.52 ± 0.47	0.296	0.47 ± 0.50	0.590
**sEV**								
C5a	8.73 ± 2.80	**0.007**	-25.97 ± 2.69	**<0.001**	7.66 ± 2.64	0.085	0.13 ± 2.85	0.061
CA125	80.37 ± 35.61	**<0.001**	-109.41 ± 34.31	**<0.001**	73.97 ± 33.69	**<0.001**	53.21 ± 36.25	**<0.001**
HE4	14.30 ± 6.66	**<0.001**	-19.39 ± 6.41	**0.004**	12.15 ± 6.30	**<0.001**	12.13 ± 6.78	**<0.001**

^*^All of the mean difference data are presented as the mean ± sd. ^†^The mean difference refers to the difference of the early-stage EOC group minus the respective group. Statistically significant differences are shown in bold. EOC. epithelial ovarian carcinoma; sEV, small extracellular vesicles; early stage, early-stage EOC group; late stage, late-stage EOC group; healthy, healthy subject group; GI cancer, gastrointestinal cancer group; ROMA, risk of ovarian malignancy algorithm.

### Serum sEV Protein Levels Differ With EOC FIGO Stage

With the observation of the difference of serum sEV proteins between early- and late-stage groups, we further examined serum sEV protein levels among EOC FIGO stages. No protein levels differed between c I and II patient groups (**Table 5**). Serum HE4 levels, ROMA indices, and serum sEV C5a levels were significantly higher in stage III patients compared to stage I and II patients (*p* < 0.001). All of the protein levels in stage IV patients significantly increased compared to other stages, except that ROMA indices, serum HE4 levels, and serum sEV C5a levels showed no difference between stage III and stage IV patients.

**Table 5:** Serum protein and sEV protein levels in the EOC FIGO stages.

	Stage I	Stage II		Stage III		Stage IV	
	Mean ± SD	Mean ± SD	*P*-value	Mean ± SD	*P*-value	Mean ± SD	*P*-value
**Serum**							
CA125	243.77 ± 360.43	67.45 ± 100.69	1.000^*^	335.05 ± 445.98	1.000^*^	1026.1 ± 1714.07	0.012^*^
					1.000^‡^		**0.002^‡^**
							**0.022^#^**
HE4	200.25 ± 318.99	224.99 ± 349.82	1.000^*^	418.59 ± 451.61	0.003^*^	540.11 ± 489.96	0.002^*^
					0.055^‡^		**0.013^‡^**
							1.000^#^
ROMA	3.58 ± 3.09	3.59 ± 3.54	1.000^*^	6.5 ± 3.26	<0.001^*^	8.52 ± 2.17	<0.001^*^
					<0.001^‡^		**<0.001^‡^**
							0.128^#^
**sEV**							
C5a	18.02 ± 27.71	14.08 ± 10.39	1.000^*^	42.13 ± 21.35	<0.001^*^	43.72 ± 21.76	<0.001^*^
					**<0.001^‡^**		**<0.001^‡^**
							1.000^#^
CA125	100.38 ± 185.13	55.28 ± 146.66	1.000^*^	148.24 ± 287.45	1.000^*^	387.56 ± 446.33	<0.001^*^
					0.465^‡^		**<0.001^‡^**
							**<0.001^#^**
HE4	16.26 ± 25.29	18.49 ± 26.03	1.000^*^	29.53 ± 47.39	0.826^*^	68.1 ± 118.35	<0.001^*^
					1.000^‡^		**<0.001^‡^**
							**0.004^#^**

^*^Statistical analysis of protein levels between patients and stage I patients. ^‡^Statistical analysis of protein levels between patients and patients with stage I and II of EOC. ^#^Statistical analysis of protein levels between stage IV patients and other stage patients. All of the data are presented as the mean ± sd. Statistically significant differences are shown in bold. ROMA, risk of ovarian malignancy algorithm; sEV, small extracellular vesicles.

### Diagnosis of EOC Using Serum sEV Markers

**Figure 1:**
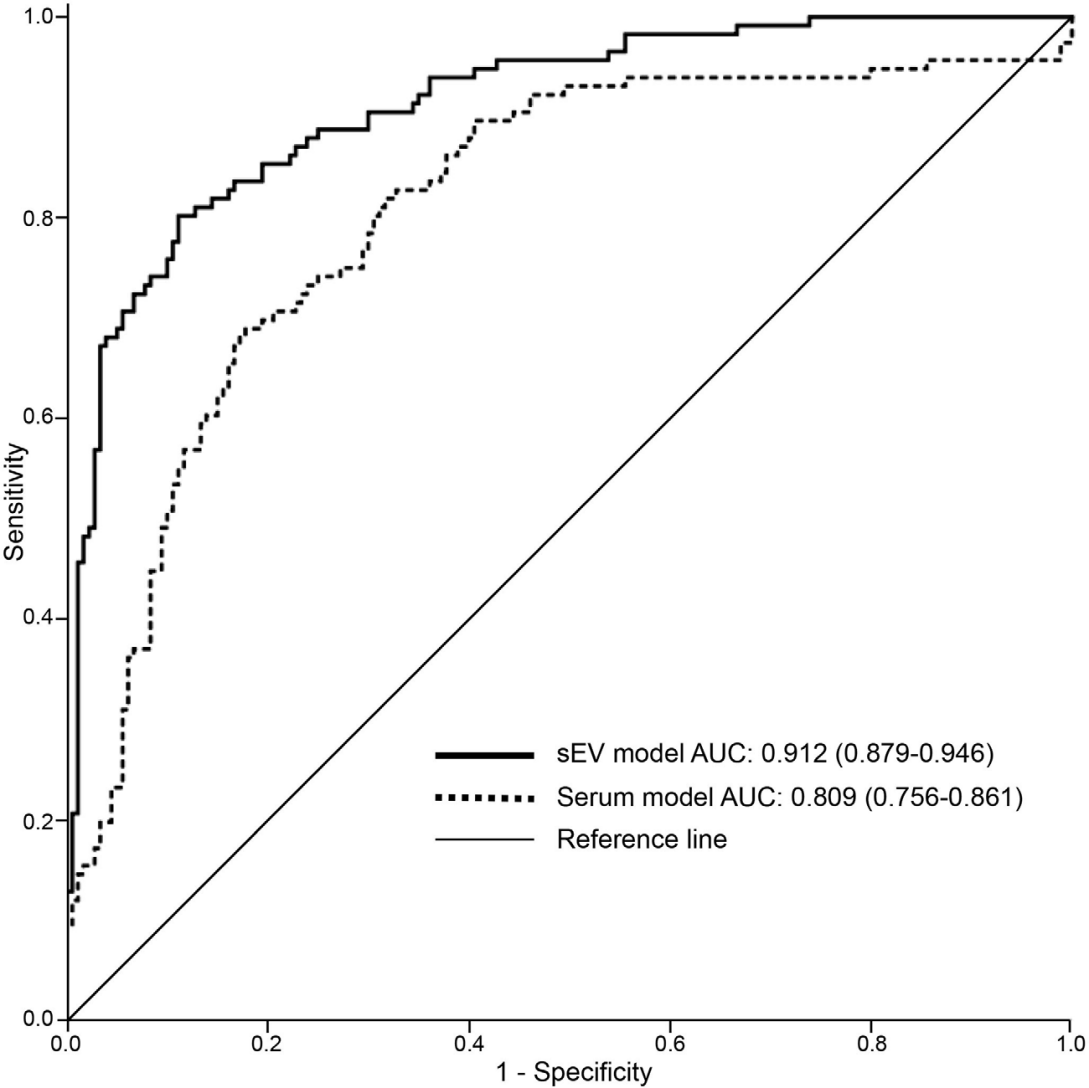
Receiver operating characteristic (ROC) analysis for identifying EOC using serum sEV or serum marker panel models. The serum sEV model consists of serum sEV levels of C5a, CA125, and HE4. Serum model includes serum level of CA125 and HE4 levels.

The predicted probability of combined serum sEV levels of CA125, HE4, and C5a (serum sEV model) as well as combined serum CA125 and HE4 (serum model) were used for ROC analysis. The area under curve (AUC) of ROC analysis of the serum sEV model (0.912) was greater than the serum model (0.809) (**Figure 1**). This indicated that the serum sEV model has better diagnosis performance compared to the combined serum model. The algorithm of serum sEV model is presented below.

$Y=0.868^{*}LN(EV\_C5a)+0.582^{*}LN(EV\_CA125)+0.933^{*}LN(EV\_HE4)-5.290$

Predicted probability = EXP(Y)/(EXP(Y) + 1) (Range: 0 to 1).

When the cutoff point was 0.370, the corresponding sensitivity was 0.80 and specificity was 0.89 (**Table 3**). The serum sEV model demonstrated much better diagnosis performance than serum marker alone or ROMA index.

The potential of serum sEV markers to identify early-stage EOC patients was further evaluated. The population was further narrowed down to early-stage EOC patients, healthy subjects, and benign ovarian cancer patients. With the calculated predicted probability above, the AUC of ROC to distinguish EOC patients was 0.888 (**Figure S3**). When the cutoff was set at 0.154, the sensitivity and specificity were 0.88 and 0.74, respectively (**Table 6**). The performance of the serum sEV model was better than ROMA, serum CA125, and serum HE4 at the set cutoff point. Even when the cutoff of serum sEV model remained at 0.370, the sensitivity and specificity were 0.58 and 0.68 (**Table 6**), respectively, which were still better than the ROMA index and serum markers.

**Table 6:** The sensitivity and specificity of serum markers and sEV model to identify the early stage EOC patients from controls.

	Sensitivity	Specificity	PPV (%)	NPV (%)
serum HE4 (Overall)*	0.32	0.74	32	74
serum HE4 (pre-/post-menopause)‡	0.29	0.63	19	75
serum CA125^#^	0.50	0.65	36	77
ROMA**	0.40	0.62	24	77
sEV model (cut-off at 0.370)^	0.58	0.68	83	85
sEV model (cut-off at 0.154)^^	0.88	0.74	57	94

^*^the cut-off was at 105.10 pmol/L; ^‡^the cut-off of pre-menopause was at 68.96 pmol/L, post-menopause was at 114.90 pmol/L; ^#^the cut-off was at 35 U/mL;^ **^the cut-off of pre-menopause was at 1.14, post-menopause was at 2.99; ^^^the cut-off was at 0.370; ^^, the cut-off was at 0.154. PPV, positive predictive value; NPV, negative predictive value; sEV, small extracellular vesicles; ROMA, risk of ovarian malignancy algorithm.

### Serum sEV Protein Levels Decrease After Operation

To assess the correlation between serum sEV protein markers and tumor burden, we compared the serum sEV levels of C5a, CA125, and HE4 of 20 patient samples prior to surgery and postoperation. All three serum sEV markers significantly decreased after cytoreductive surgery (**Table 7**). This is suggestive of the positive correlation between tumor burden and serum sEV markers.

**Table 7:** Comparison of serum sEV protein levels before and after cytoreductive surgery.

sEV	Pre-operation	Post-operation	*P*-value
C5a	20.88 ± 23.02	11.31 ± 8.48	**0.044**
CA125	226.71 ± 254.05	97.1 ± 129.9	**0.008**
HE4	40.11 ± 65.58	6.06 ± 4.19	**0.025**

All of the data are presented as the mean ± sd. Statistically significant differences are shown in bold. sEV, small extracellular vesicles.

## Discussion

The survival rate of EOC is closely related to the stage at diagnosis, with those diagnosed at the earlier stage with a greater chance of survival (23). In this study, we have established a model using the levels of CA125, HE4, and C5a in serum sEVs to identify EOC patients and early-stage EOC patients from subjects with benign ovarian diseases or other diseases. This novel serum sEV model has been compared with the current serum protein marker HE4 and CA125, as well as ROMA, which was approved by the U.S. FDA (510(k) Number: k103358). The results have demonstrated that serum sEV model is much better diagnosis performance than serum marker alone or ROMA index in the diagnosis of EOC patients, including early-stage EOC. This provides the possibility of the levels of serum sEV proteins for the diagnosis of early-stage EOC.

Several studies have explored the potential of serum sEV content (e.g., mRNA, miRNA, protein) as biomarkers for ovarian cancer (27, 28). These studies mainly employed ultracentrifugation to isolate serum sEVs, which may be regarded as the “gold standard” of serum sEV isolation (26). However, ultracentrifugation often involves a large volume of plasma or serum, hours of spinning in a vacuum and strict temperature control, costly equipment, and maintenance. This is thus challenging to implement for clinical utilization. On the contrary, in this study, we adapted a polyethylene glycol (PEG)-based approach to isolate serum sEVs (24), which has advantages of being high-throughput, with better reproducibility, and lower cost.

Over the years, several tests have been developed to identify malignant ovarian cancer, including serologic markers (e.g., CA125 and HE4), ultrasonography, imaging, and combined multimodalities. Transvaginal ultrasonography (TVUS) (29) and computed tomography (CT) (30) have been proven to be inefficient in screening early-stage EOC, which may further be underutilized in less developed clinical circumstances such as poor staffing and equipment availability (31). Both the Prostate, Lung, Colorectal, and Ovarian (PLCO) (32) Cancer Screening Trial and the United Kingdom Collaborative Trial of Ovarian Cancer Screening (UKCTOS) (29) showed that screening ovarian cancer in asymptomatic women using serum CA125 levels and/or TVUS has brought little benefit. Based on its cost-effectiveness and demographic coverage, biomarkers have the highest potential for the early diagnosis and screening of EOC (31).

Serum levels of CA125 and HE4 are both well-established biomarkers for ovarian cancer. So far, both markers are only intended to monitor the progress and estimate the treatment efficacy of EOC. However, the levels of these two proteins in serum sEVs showed better capability of distinguishing EOC patients from non-EOC patients. The increase in serum sEV CA125 and HE4 levels in EOC patients is coincided with the findings of previous proteomics studies (33).

The discovery of novel serological protein biomarkers mainly relies on mass spectrometry and proteomics. During the discovery phase of proteomics, the bona fide candidate markers are masked by high-abundance proteins (e.g., albumin and immunoglobulins), which account for over 99% of the total proteins. However, the percentage of these abundant proteins is significantly reduced in extracellular vesicles (16), which makes serum sEVs a potential source for biomarker discovery. In our previous study, complementary-, coagulation-, apoptosis-related pathways were discovered to be significantly overexpressed in serum sEVs of EOC patients (20).

Our results on ROMA indices are discordant to those of previous studies (34) and may be attributed to three causes. First, the Chinese population showed nearly 30% lower serum HE4 levels (overall cut-off: 105.10 pmol/L) compared to Caucasians (overall cutoff: 140.00 pmol/L) (15). In addition, neither the algorithm nor the cutoff for the ROMA index has been adjusted for the Chinese population. Second, the U.S. Food and Drug Administration (FDA) clearances of serum CA125 II (510(k) Number K143534) (35) and HE4 (510(k) Number: k112624) (36) are employed to aid the detection of residual or recurrent ovarian carcinoma and to monitor disease progression or response to therapy. ROMA (510(k) Number: k103358) (37) was approved by the US FDA for “assessing whether a premenopausal or postmenopausal woman who presents with an ovarian adnexal mass is at high or low likelihood of finding malignancy on surgery”. However, owing to lack of evidence of clinical trials on the Chinese population, this intended use has not been approved by the National Medical Products Administration. Finally, the cohort composition of the present study is different. The Roche ROMA cohort consists of approximately 80% benign ovarian diseased patients and 10% EOC (about 20% early stage and 80% late stage), but this cohort comprised approximately 40% EOC (about 40% early stage and 60% late stage) and 25% benign ovarian diseased patients (34, 37).

The mechanism of the progression of EOC remains unclear. With the concurrence of multiple foci and carcinomatosis after ovary-removal surgery (38), detection of EOC or postoperative recurrence at the early stage is challenging using non-invasive imaging tools. Although this serum sEV protein marker panel can detect EOC better at the early stages, there is still a need to establish a marker panel for early diagnosis or even as a screening tool for EOC patients. Therefore, more markers need to be discovered and validated for better marker panels not only to screen EOC but also to reveal the underlying mechanism of EOC.

## Conclusion

In summary, our study disclosed that CA125, HE4, and C5a levels in serum sEVs can identify EOC at the early stage. In spite of the complexity of clinical diseases, comparison of serum protein marker alone or ROMA index using a multi-center population-based study in the Chinese population confirmed that serum sEV model has relatively high performance for the diagnosis of early-stage EOC. Further test this model in an even larger and more complex population to determine its performance and limitations is required.

## Data Availability Statement

The original contributions presented in the study are included in the article/**Supplementary Material**. Further inquiries can be directed to the corresponding authors.

## Ethics Statement

This study has received approval from the Ethics Committee of Tianjin Central Hospital of Gynecology Obstetrics (IRB#2018KY002), Fudan University Shanghai Cancer Center (IRB#1909182-4), the Third Affiliated Hospital of Kunming Medical University (IRB#2016NS089), the Third Affiliated Hospital of Guangzhou Medical University (IRB#2018CYFYHEC-021-02), and the First Affiliated Hospital of Chengdu Medical College [IRB#201807(3)]. The patients/participants provided their written informed consent to participate in this study.

## Author Contributions

YH, YaZ, HW, and DZ conceived and designed the study. PL, YB, BS, WZ, ZL, YiZ, XX, QC, XS, XD, and ZG performed the experiments. PL, YB, BS, YaZ, and DZ contributed to the data analysis. PL, BS, DZ, YaZ, HW, and YH wrote and revised the manuscript All authors contributed to the article and approved the submitted version.

## Conflict of Interest

Authors YB, ZL, XX, DZ, and YaZ were employed by 3D Medicines.

The remaining authors declare that the research was conducted in the absence of any commercial or financial relationships that could be construed as a potential conflict of interest.

## Publisher’s Note

All claims expressed in this article are solely those of the authors and do not necessarily represent those of their affiliated organizations, or those of the publisher, the editors and the reviewers. Any product that may be evaluated in this article, or claim that may be made by its manufacturer, is not guaranteed or endorsed by the publisher.
