# Supplementary material for: Exploration of Potential Diagnostic Value of Protein Content in Serum Small Extracellular Vesicles for Early-Stage Epithelial Ovarian Carcinoma
Source: Front Oncol. 2021 Sep 15;11:707658. doi: 10.3389/fonc.2021.707658 (PMC8479155; doi:10.3389/fonc.2021.707658)
Supplement: Supplementary file 1 [file DataSheet_1.pdf]

Supplementary Material

Figure S1

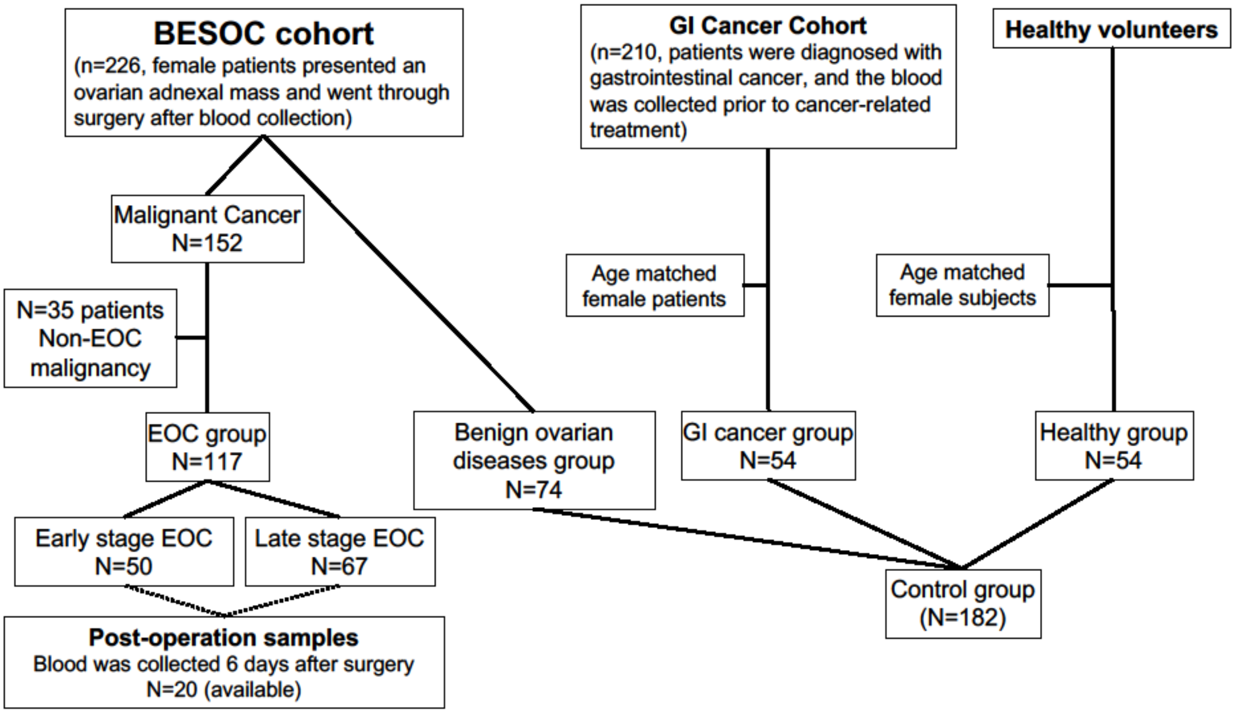

Figure S1. Flowchart of the structure of the clinical samples. In this study, 299 subjects were enrolled. In the event group, 117 patients were adjudicated as EOC, while 74 patients with benign ovarian diseases were included. In addition, 54 apparently healthy subjects, and 54 patients with gastrointestinal cancer were also used as controls. Abbreviations: BESOC: Biomarker for Early Stage Ovarian Cancer; EOC: epithelial ovarian carcinoma; GI cancer: gastrointestinal cancer.

Figure S2

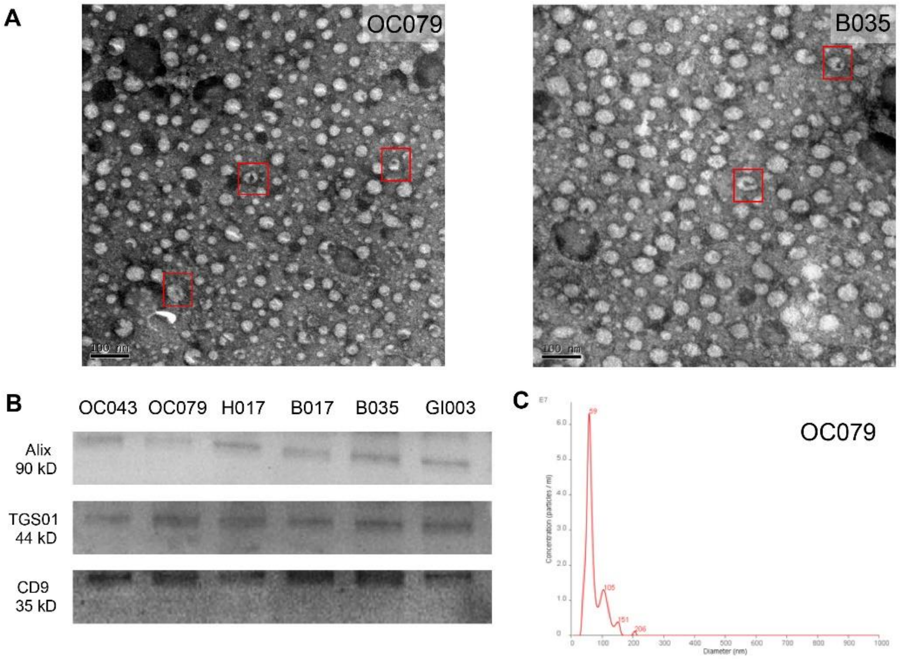

Figure S2. Characteristic proteins and morphology of small extracellular vesicles (sEVs). (A) Images of sEVs from two representative samples were taken by scanning electron microscopic analysis. The representative sEVs morphology is highlighted by a red box. (B) The protein levels of Alix, TSG101, and CD9 in the sEVs of 6 representative samples were stained using western blotting. OC043 was a late stage EOC sample; OC079 was an early stage EOC sample; H017 was a healthy subject sample; B017 and B035 were from benign ovarian disease group; and GI003 was from gastrointestinal cancer group. (C). Nanoparticle tracking analysis results from representative sEVs samples are shown.

Figure S3

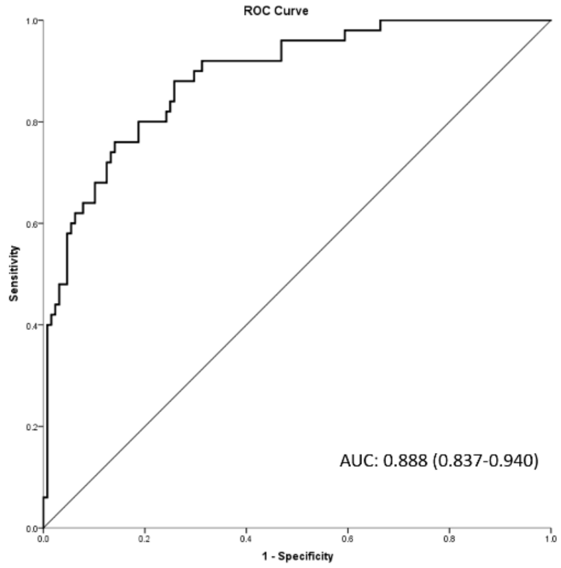

Figure S3. Receiver operating characteristic (ROC) analysis for identifying early stage EOC patients from control cohort using serum sEV model. The serum sEV model includes serum sEV levels of C5a, CA125, and HE4 to identify EOC patients for early stage.
